# ID4 Promotes Breast Cancer Chemotherapy Resistance via CBF1-MRP1 Pathway

**DOI:** 10.7150/jca.31988

**Published:** 2020-04-06

**Authors:** Xi Zhang, Guangyan Gu, Lin Song, Dan Wang, Yali Xu, Shuping Yang, Bin Xu, Zhixin Cao, Chunmei Liu, Chunming Zhao, Yuanyuan Zong, Yejun Qin, Jiawen Xu

**Affiliations:** 1 Department of Breast and Thyroid Surgery, Shandong Provincial Hospital Affiliated to Shandong First Medical University, Shandong, 250021, PR China; 2 Department of Breast and Thyroid Surgery, Shandong Provincial Hospital Affiliated to Shandong University, Jinan, Shandong, 250021, PR China; 3 Department of Histology and Embryology, Shandong University Cheeloo College of Medicine, Jinan, 250012, Shandong, China; 4 Department of Pathology, Shandong Provincial Hospital Affiliated to Shandong First Medical University, Shandong, 250021, PR China; 5 Department of Pathology, Shandong Provincial Hospital Affiliated to Shandong University, Jinan, Shandong, 250021, PR China; 6 Department of Science and education, Shandong Provincial Hospital Affiliated to Shandong First Medical University, Shandong, 250021, PR China; 7 Department of Science and education, Shandong Provincial Hospital Affiliated to Shandong University, Jinan, Shandong, 250021, PR China; 8 Department of Oncology, Shandong Provincial Hospital Affiliated to Shandong First Medical University, Shandong, 250021, PR China; 9 Department of Oncology, Shandong Provincial Hospital Affiliated to Shandong University, Jinan, Shandong, 250021, PR China; 10 Department of Pathology, Shengli Oil Field Central Hospital, Dongying, Shandong Province, 257034, P.R China; 11 Department of Clinical Laboratory, Shandong Provincial Hospital Affiliated to Shandong First Medical University, Shandong, 250021, PR China; 12 Department of Clinical Laboratory, Shandong Provincial Hospital Affiliated to Shandong University, Jinan, Shandong, 250021, PR China; 13 Department of Ophthalmology, Shandong Provincial Hospital Affiliated to Shandong First Medical University, Shandong, 250021, PR China; 14 Department of Ophthalmology, Shandong Provincial Hospital Affiliated to Shandong University, Jinan, Shandong, 250021, PR China

**Keywords:** ID4, triple negative breast cancer, chemotherapy resistance, CBF1, MRP1

## Abstract

Chemo-resistance is considered a key problem in triple negative breast cancer (TNBC) chemotherapy and as such, an urgent need exists to identify its exact mechanisms. Inhibitor of DNA binding factor 4 (ID4) was reported to play diverse roles in different breast cancer molecular phenotypes. In addition, ID4 was associated with mammary carcinoma drug resistance however its functions and contributions remain insufficiently defined. The expression of ID4 in MCF-7, MCF-7/Adr and MDA-MB-231 breast cancer cell lines and patients' tissues were detected by RT-PCR, western blot and immunohistochemistry. Furthermore, TCGA database was applied to confirm these results. Edu and CCK8 assay were performed to detect the proliferation and drug resistance in breast cancer cell lines. Transwell and scratch migration assay were used to detected metastasis. Western blot, TCGA database, Immunoprecipitation (IP), Chromatin Immunoprecipitation (ChIP) and Luciferase reporter assay were used to investigate the tumor promotion mechanisms of ID4. In this study, we report that the expression levels of ID4 appeared to correlate with breast cancers subtype differentiation biomarkers (including ER, PR) and chemo-resistance related proteins (including MRP1, ABCG2, P-gp). Down-regulation of ID4 in MCF-7/Adr and MDA-MB-231 breast cancer cell lines significantly suppressed cell proliferation and invasion, however enhanced Adriamycin sensitivity. We further demonstrated that the oncogenic and chemo-resistant effects of ID4 could be mediated by binding to CBF1 promoter region though combination with MyoD1, and then the downstream target MRP1 could be activated. We reveal for the first time that ID4 performs its function via a CBF1-MRP1 signaling axis, and this finding provides a novel perspective to find potential therapeutic targets for breast cancer chemotherapy.

## Background

Breast cancers are regarded as the most prevalent malignant carcinoma and the second leading cause of cancer death in women 1. Breast cancer is divided into four main phenotypes according to the expression of estrogen receptor (ER), progesterone receptor (PR) and human epidermal growth factor receptor-2 (HER2): Luminal A, Luminal B, Her-2 overexpression and triple negative breast cancer (TNBC) 2. Among these four types, TNBC is associated with very high rates of recurrence and chemotherapy resistance 3. Despite advances in oncology to optimize treatment options, the prognosis of TNBC patients still remains poor, partially due to the lack of targeted therapy 4, 5. One of the main mechanisms of drug resistance is high expression of ATP-binding cassette (ABC) transporters such as P-gp, MRP1 and ABCG2, which transport various molecules across extra- and intra- cellular membranes 6, 7. It is in an urgent need therefore to identify novel strategies and potential therapeutic targets for early diagnostics and therapeutics of breast cancers, especially TNBC.

Inhibitor of DNA-binding (ID) proteins belong to a family of four helix-loop-helix transcriptional regulators which are considered as the key regulators of development and tumorigenesis 8, 9. The new member of this family, ID4, controls tumor progress of different cancers by regulating upstream of key developmental pathways 10-12. There is still disagreement surrounding how ID functions in treating breast cancers. The down-regulation of ID4 expression in ER+ breast cancers was observed, indicating the inhibition effect of ID4 13. According to Nasif's report, ID4 was significantly silenced by promoter methylation in ER+ breast cancers and overexpression of ID4 in ER+ cell lines could result in decreased migration capacity and reduced number of colonies, functioning as a tumor suppressor gene 14. However, Baker et al identified the negative relationship between ID4 and BRCA1 15, 16, and Branham confirmed the notion and unmethylation status of ID4 was a highly effective predictor for BRCAness tumors 17. Interestingly, it was also reported that the up-regulation of ID4 promoted the proliferation of ER- SKBr3 cells 18. Thus, the role of ID4 in breast cancer is not clear where both an oncogenic and a tumor suppressor function have been attributed. In addition, it was proposed ID4 could participate in the chemoresistance process. In non-small cell lung cancer, ID4 inhibited the production of cisplatin by activating the p38-MAPK pathway 19, 20. The over-expression of ID4 in glioma stem cells directly inhibited the expression of microRNA-9 by inhibiting the expression of SOX2, which in turn regulated ATP-binding cassette (ABC) transporters, finally leading to less sensitivity to nitrosourea drugs in cancer cells 21. However, it is still unknown whether ID4 can mediate tumor cell sensitivity to chemotherapeutic drugs in breast carcinoma, especially TNBC.

The Notch pathway is a highly conserved cell signaling pathway that plays a pivotal role in a variety of cellular processes, including proliferation, invasiveness and drug resistance 22, 23. Moreover, Notch1 is critical in cell fate decisions in the developing nervous system and several cancer cells, whist its activation is also involved in chemoresistance processing 24, 25. It is reported that enforced ID4 function can drive the activation of the Notch1 pathway in brain tumor cells 11. However, it is unknown whether ID4 affected Notch signaling in breast cancer. In this study, we demonstrated that ID4 regulates the drug resistance of breast cancer by connecting with Notch1 in a new way.

In an effort to discover novel ID4 functions in this study, we firstly detected the expression of ID4 in 100 breast cancer tissues after surgery and analyzed the correlation between the ID4 and breast cancer phenotype markers and some ABC transporters. In addition, knockdown of ID4 in TNBC breast cancer lines significantly suppressed the proliferation, migration, invasion and Adriamycin resistance *in vivo* or *in vitro*. Furthermore, we identified CBF1 as a potential downstream mediator of the effects mentioned above. Result showed that ID4 may target the CBF1 pathway by directly binding to its promoter region via combination with MyoD1, therefore CBF1 activated the function of MRP1 to enhance the chemotherapy resistance in breast cancers.

## Materials and Methods

### Clinical specimens

The 100 cases of invasive breast cancer specimens were collected form the Department of Pathology of Shandong provincial hospital affiliated to Shandong First Medical University between 2011 and 2013. The diagnoses were reviewed by two pathologists based on histology, clinical information and other related data. Informed consent was obtained from all the participants of this study, and the use of tissue specimens was approved by the Research Ethics Committee of Shandong Provincial Hospital Affiliated to Shandong First Medical University (Jinan, China).

### Cell lines

Breast cancer cell lines(MCF-7, MCF-7/Adr and MDA-MB-231) were obtained from Shanghai Institutes for Biological Sciences Cell Resource Center (Shanghai, China), and cultured in Dulbecco's modified Eagle's medium (DMEM, high glucose; Gibco-BRL, Grand Island, USA) supplemented with 12% fetal bovine serum, 100 units/mL penicillin and 100μg/mL streptomycin. The cells were cultured at 37℃ in a humidified atmosphere containing 5% CO_2_.

### shRNA interference and Lentiviral-vector infection

The vector GV248 carrying expressing a silencing form of ID4(sequences: 5'-CCCAACAAGAAAGUCAGCATT-3'),U6-MCS-Ubiquitin-EGFP-IRES-puromycin, was constructed (GeneChem Co., Shanghai, China), and the extracion and purification were followed by the protocol of Plasmid Extraction Kit provided by QIAGEN. MCF-7/Adr and MDA-MB-231 cells (1×10^5^ cells/ml) were seeded in 12-well plates in triplicates and were transfected with shRNA targeting ID4 after overnight incubation, Lipofectaminetamine® 2000 (Invitrogen, Carlsbad, CA USA) and Opti-MEM® (Gibco, New York, USA) were used for transfection according to the manufacturer's introduction. The knowdown efficiency was validated by qRT-PCR (for RNA levels after 24h incubation) and western blot (for protein levels after 48h incubation, data not shown), respectively. Afterward, then the ID4-shRNA plasmid was packaged into GV248 lentivirus vector according to the protocol of Lenti- easy Packaging System (Lot: LPK001, Genechem, Shanghai). The cells were planted into 6-well plates, and add polybrene to a final concentration of 5 μg/ml. Finally, add 20 μl lentivirus solution with titer of 1×10^8^ TU/ml. An empty lentiviral vector (GeneChem Co., Shanghai, China) was used as a negative control. The knowdown efficiency was validated by qRT-PCR and western blot (data not shown).

### Immunohistochemistry

The methods of Immunohistochemistry were similar to our previous studies. For the immunohistochemical analysis of ID4 (Cat.# ab49261, Abcam, USA), ER (Cat.# ab75635, Abcam, USA), PR (Cat.# ab32085, Abcam, USA), Her-2 (Cat.# 3B5, Abcam, USA), Ki-67 (Cat.# OTI5D7, Abcam, USA), MRP1 (Cat.# ab24102, Abcam, USA), ABCG2 (Cat.# ab24115, Abcam, USA), P-gp (Cat.# ab103477, Abcam, USA), 4 mm-thick sections from the formalin-fixed, paraffin-embedded tissues were used. The samples were deparaffinized in xylene and rehydrated through a graded series of ethanol washes. After the endogenous peroxidase was inhibited and the antigen was retrieved (microwave irradiation in 0.01 M citrate buffer at pH 6.0), the sections were incubated with primary antibody at 4℃ overnight and then with horseradish peroxidase (HRP)-conjugated secondary antibodies (DakoCytomation, Denmark). After washing, tissues were stained for 5 min with 3,30-diaminobenzidine (DAB) chromogen and counterstained with hematoxylin (Zhongshan Golden Bridge,Inc), dehydrated and mounted on cover slips. Negative controls were treated without the primary antibody.

### Evaluation of immunohistochemical stained samples

For ID4 (nuclear staining), Ki-67(nuclear staining), MRP1(membrane staining), ABCG2 (membrane staining) and P-gp (membrane staining) immunohistochemical evaluation, a staining score value was calculated as the intensity of staining (negative: 0, weak:1, moderate:2, strong:3) multiplied by the percentage of positive tumor cells (<5%:0, 6%-25%: 1, 26%-50%: 2, 50%-75%: 3,>75%:4). A final staining score of less than 3 was regarded as low expression, and the score between 4 and 12 was regarded as high expression. The expression of ER and PR was scored as “negative” (nuclear staining in < 1% of tumor cells) or “positive” (nuclear staining in ≥ 1% of tumor cells). The expression of HER-2 was scored as “negative” (no reactivity or membrane staining in < 10% of tumor cells), “1+” (faint/barely perceptible staining in > 10% of tumor cells), “2+” (weak to moderate membrane staining in > 10% of tumor cells), and “3+” (uniform intense membrane staining of > 10% of invasive tumor cells). Samples giving a result of 2+ were retested using fluorescence in situ hybridization (FISH). Samples were determined to be positive for HER-2 if the immunohistochemistry score was 3+ or the FISH amplified ratio of HER-2 to CEP17 was greater than 2.

### Immunoblotting

Proteins were separated by 10% SDS-PAGE and transferred to polyvinylidene fluoride membranes (Millipore, Bedford, A, USA). After blocking in 5% nonfat milk for 1 h at room temperature, the membranes were incubated overnight with primary antibodies against ID4 (Cat.# ab49261, Abcam, USA), Notch1 (Cat.# ab52627, Abcam, USA), CBF1 (Cat.# ab180588, Abcam, USA), Hes1 (Cat.# 108937, Abcam, USA), JAG1 (Cat.#ab109536, Abcam, USA), MRP1 (Cat.# ab24102, Abcam, USA), ABCG2 (Cat.# ab24115, Abcam, USA), P-gp (Cat.#ab103477, Abcam, USA) and GAPDH (Cat.#ab8245, Abcam, USA) at 4℃. Then, the membranes were incubated for 1 h at 4℃ with the appropriate HRP-conjugated secondary antibodies (Cat. #7074, Cell Signaling, Beverly, MA, USA). Protein expression levels were detected via enzyme-linked chemiluminescence (Pierce, Rockford, USA).

### Cell proliferation assay

Breast cancer cells were planted in 96-well plates at a density of 2,500 cells per well for 24 hours. EDU immunocytochemistry staining was performed with Cell-Light™ EdU Apollo In-Vitro Imaging kits (Ribobio, Guangzhou, China).For cell viability, 2,500 cells per well were seeded in 96-well plates and the absorbance results were evaluated with Cell Counting Kit-8 (DOJINDO, Japan) by testing one, two and three days after cells were plated to confirm the identical cell number of each group respectively (according to the manufacturer's instruction).

### Adriamycin sensitivity assay

The MDA-MB-231 and MCF-7/Adr cells were treated with different dose of Adriamycin. After 24h, CCK8 assay was performed to detect the cell viability. Cell viability was assigned using the following formula:


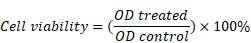


### Cell invasion/migration assay

Breast cancer cells were cultured at about 80% confluence. Cells were starved in basal medium without fetal bovine serum for 16h. Matrigel cell invasion assay was carried out using the BD BioCoat Tumor Invasion System (BD Biosciences #354165) as recommended by the manufacturer. 5×10^4^ starved mammary carcinoma cells were seeded into the apical chambers, followed by adding a chemoattractant (basal medium plus 10% FBS) to the basal chambers. After 24h incubation, cells in the upper chambers were carefully removed with a cotton swab and the cells that had traversed the membrane were fixed in methanol and stained with leucocrystal violet. The number of invasive cells was determined by counting the leucocrystal violet stained cells. For quantification, cells were counted under a microscope in five fields (up, down, median, left, right. ×200). For the migration assay, 5×10^4^ starved cells were seeded in serum-free medium in the upper chamber. After 12 h incubation at 37°C, cells in the upper chamber were carefully removed with a cotton swab and the cells that had traversed the membrane were fixed in methanol and stained with leucocrystal violet. Migration cells were counted under a microscope in five fields (up, down, median, left, right. ×200).

### Co-immunoprecipitation (Co-IP) assays

1500ug of MCF7/Adr protein of precleared cell lysates were immunoprecipitated with 1 µg primary antibodies against MyoD1 (Cat.# ab126726, Abcam, USA) by overnight incubations at 4°C. The immune complexes were pre-cipitated with Protein A Sepharose CL-4B (Amersham, Piscat-away, N.J., USA) and resolved by SDS-PAGE. The bound proteins were then detected using primary antibodies ID4 and MyoD1 by western blot analysis.

### Chromatin immunoprecipitation (CHIP) assays

MCF-7/Adr cells were cross-linked with 1% PFA and quenched by adding 125 mM glycine. Chromatin was isolated by addition of cell lysis buffer (1% SDS, 10 mM EDTA, 50 mM Tris-HCl, pH 8.1, 1 mM PMSF) and sheared to fragments of 300-500 bp by sonication. Lysates were pre-cleared for 1-2 hours using Salmon Sperm DNA/Protein Agarose (EMD Millipore, Billerica, MA, USA), after which precipitation was induced using anti-MyoD1 (Cat.# ab126726, Abcam, USA). An isotype matched IgG was used as a negative control. To then reverse the DNA cross-linking, the precipitates were incubated with pronase for 2 h at 42°C and 68°C for 8 h. The promoter DNA in the precipitates was detected by qRT-PCR.

### Quantitative reverse transcription PCR (qRT-PCR)

Total RNA was extracted using an RNA Isolation Plus kit (Cat. #9108, Takara, Dalian, China) according to the manufacturer's instructions. The extracted RNA was then reverse transcribed to cDNA using PrimeScript1 RT Master Mix (Cat. # DRR036A, Takara, Dalian, China) at 37℃ for 15 min, 85℃ for 5s, and then 4℃. qPCR was performed in a 10 ml reaction volume using the SYBR1 PremixExTaqTM (Cat. # RR420A, Takara, Dalian, China) and ABI7900HT Real-Time PCR System (Life, Singapore). The thermal cycle conditions were: one cycle at 95℃ for 30s, 40 cycles of amplification at 95℃ for 5s, followed by 60℃ for 30s. The mRNA level was normalized to the geometric mean of GAPDH (conserved gene) mRNA to control the variability in expression levels, and the results were analyzed using the Δ ΔT method. The primer sequences are shown in Table 1.

### *In vivo* experiments

All experimental animal procedures were conducted strictly in accordance with the Guide for the Care and Use of Laboratory Animals and approved by the Animal Care and Use Committee of the Shandong provincial hospital affiliated to Shandong First Medical University. The female BALB/c nude mice were obtained from Cancer Institute of the Chinese Academy of Medical Science, which were randomized divided into three groups in a blinded manner, each group including five 4-weeks-old nude mice. For subcutaneous xenograft study, 1×10^6^cells were subcutaneously injected in the right flanks of nude mice. Adriamycin was injected through vena caudalis by 2 mg/kg/d. The tumor volume was determined using the eq. V = 0.5 × D × d2 (V, volume; D, longitudinal diameter; d, latitudinal diameter). The developing tumors were observed for 35 days.

### Statistical analysis

Quantitative data are expressed as mean ± standard deviation (SD). Graphpad Prism (Graphpad Software, San Diego, CA) was used for data analysis. The Student's T test or one-way analysis of variance (ANOVA) was used to assess significant differences between groups. The Chi-square test was used to analyze the relationship between categorical variables. P<0.05 was considered statistically significant.

## Results

### ID4 expression was increased in ER negative breast cancer and correlated with molecular phenotypes

**Figure 1:**
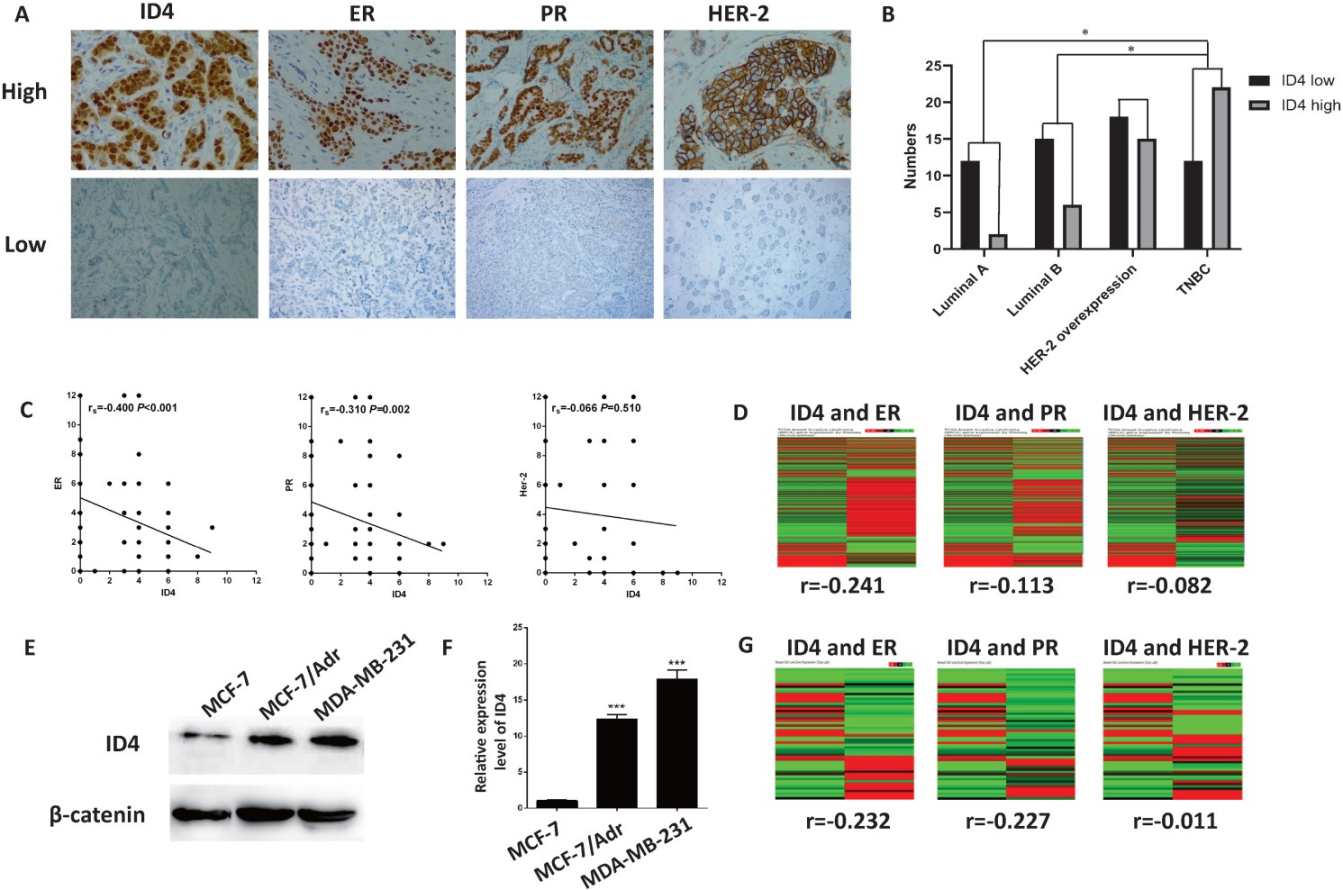
** ID4 was correlated with the molecular phenotype of breast cancer. (A)** Representative immunostaining of ID4, ER, PR and HER-2 in human breast cancer samples. ID4, ER and PR immunoreactivity were detected in the nucleus and HER-2 on the cell membrane. Magnifications were 400× in ER high and HER-2 low cases, and 200× in other cases. **(B)** ID4 was more expressed in TNBC subtype breast cancer than Luminal subtype. **(C)** IHC assay showed that ID4 expression negatively correlated with the expression of ER (r=-0.480, P<0.001) and PR (r=-0.310, P=0.002). **(D)** TCGA database showed that ID4 negatively correlated with ER (r=-0.241, P<0.001), PR (r=-0.113, P<0.001) and Her-2 (r=-0.082, P=0.004) expression in tumor tissue samples. n=1214. **(E)** Western blot result showed that the protein level of ID4 was much higher in the TNBC cell lines (MCF-7/Adr and MDA-MB-231) than in the luminal cell line MCF-7. **(F)** qRT-PCR result showed that the mRNA level of ID4 was much higher in MCF-7/Adr and MDA-MB-231 cells than in MCF-7 cells. **(G)** TCGA database showed that ID4 negatively correlated with ER (r=-0.232, P=0.092) and PR (r=-0.227, P=0.099) expression among 54 breast cancer cell lines.

We first detected the expression of ID4, ER, PR and Her-2 in 100 breast cancer tissues via immunochemistry(Fig. 1A). The result showed that ID4 expression was significantly higher in TNBC subtypes (Fig. 1B) and with analysis demonstrated negative correlations between ID4 and ER, PR (Fig. 1A). Using the TCGA data base, we found that ID4 expression was negatively correlated with the ER, PR in 1214 breast cancer tissue samples(Fig. 1D). In addition, we also tested ID4 expression in breast cancer cell lines. The results showed that the expression of ID4 was much greater in TNBC cell lines, such as MCF-7/Adr and MDA-MB-231, than in MCF-7 cell lines (Luminal subtype) (Fig. 1E, F). The expression of ID4 also negatively correlated with ER and PR expression in 54 breast cancer cell lines according to the TCGA database(Fig. 1G).

### Knockdown of ID4 inhibited the proliferation of the TNBC cell lines

**Figure 2:**
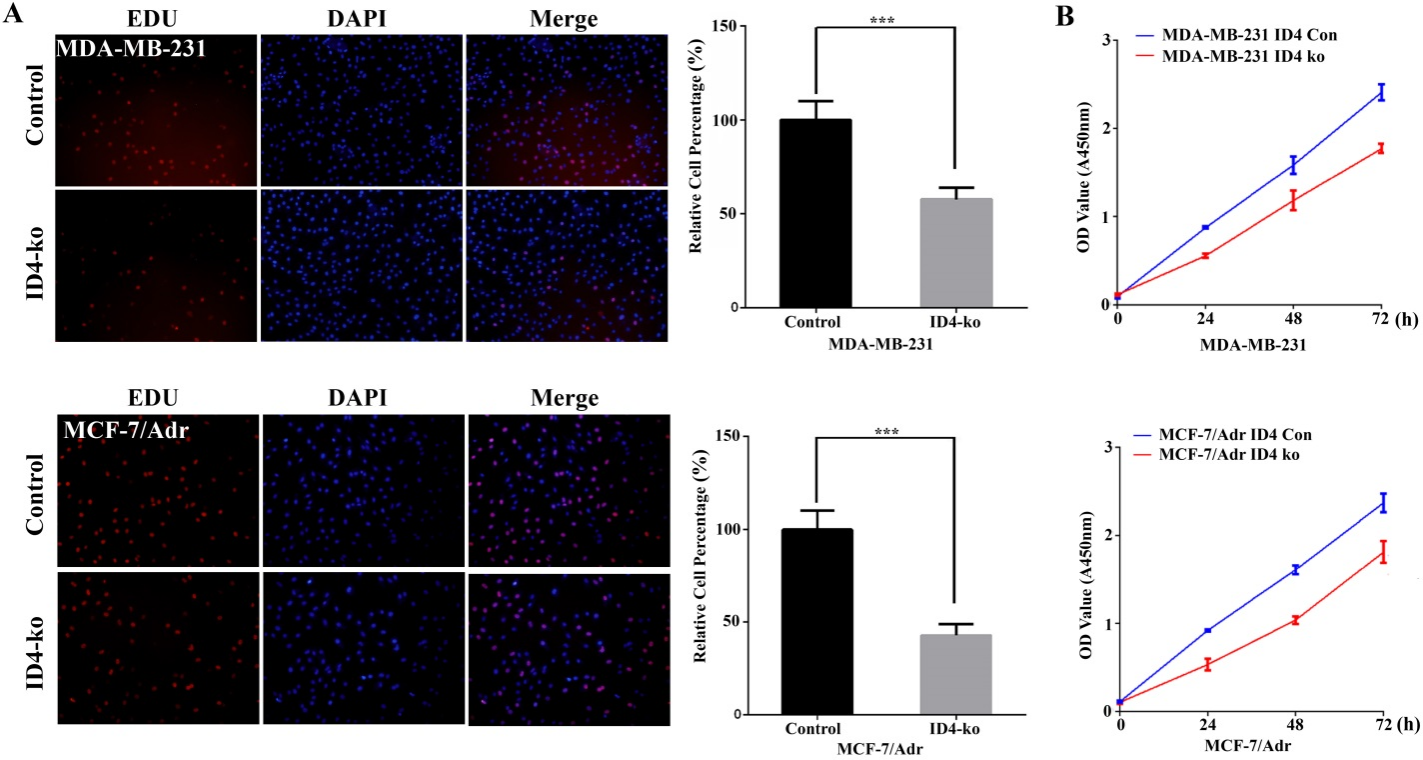
** ID4 promoted the viability of MDA-MB-231 and MCF-7/Adr cells. (A)** Cell proliferations were determined by EDU staining assay. The results represent mean ± s.d. from three independent experiments. ***P < 0.001 vs. NC. **(B)** The growth curves of the ID4 knock down MDA-MB-231 and MCF-7/Adr cells were shown using CCK8 assay. Data was presented as mean ± s.d. from three independent experiments.

To shed light on whether ID4 modulates breast cancer cell proliferation in TNBC cell lines, we infected MDB-MA-231 and MCF-7/Adr cell lines with lentiviruses expressing small hairpin RNAs (shRNAs) to knockdown ID4 expression. Lentiviral vectors with nonspecific shRNAs were taken as the negative control. qRT-PCR was used to test the knockout efficiency (data not shown). The EDU cell proliferation assay was applied to evaluate whether ID4 could affect viability of breast cancer cell. As depicted in Fig. 2A, depletion of ID4 resulted in a significant proliferation inhibition in MDB-MA-231 and MCF-7/Adr cells. CCK8 assay results also supported this phenomenon(Fig. 2B).

### Knockdown of ID4 inhibited the migration and invasion of the TNBC cell lines

**Figure 3:**
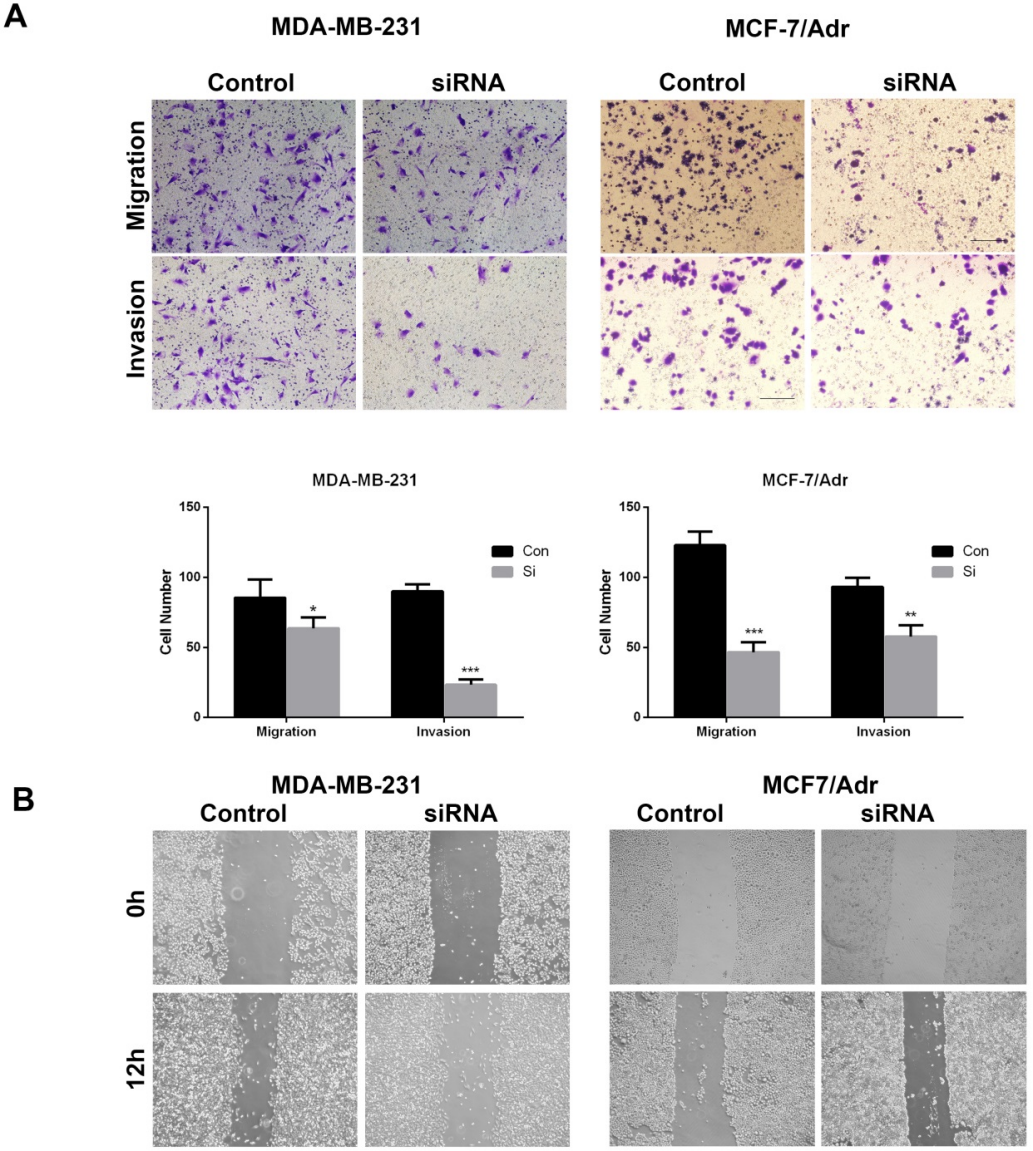
** ID4 promoted the migration/invasion of MDA-MB-231 and MCF-7/Adr cells. (A)** Cell migration and invasion was determined by transwell assay. Each bar represents mean ± s.d. from three independent experiments. **(B)** The scratch test results showed that knockdown of ID4 significantly decreased the migration ability of MDA-MB-231 and MCF-7/Adr cells.

Furthermore, a transwell assay was applied to determine whether ID4 regulated the migration and invasion of TNBC cells, with the results confirming that the number of migrating and invading MDB- MA-231 or MCF-7/Adr cells with ID4 knockdown was significantly decreased compared to that of the control cells (Fig. 3A). In addition, the results of scratch test were in line with those of transwell assay (Fig. 3B). These evidences showed that ID4 played an important role in promoting TNBC invasive properties.

### ID4 is associated with breast cancer chemo-resistance

**Figure 4:**
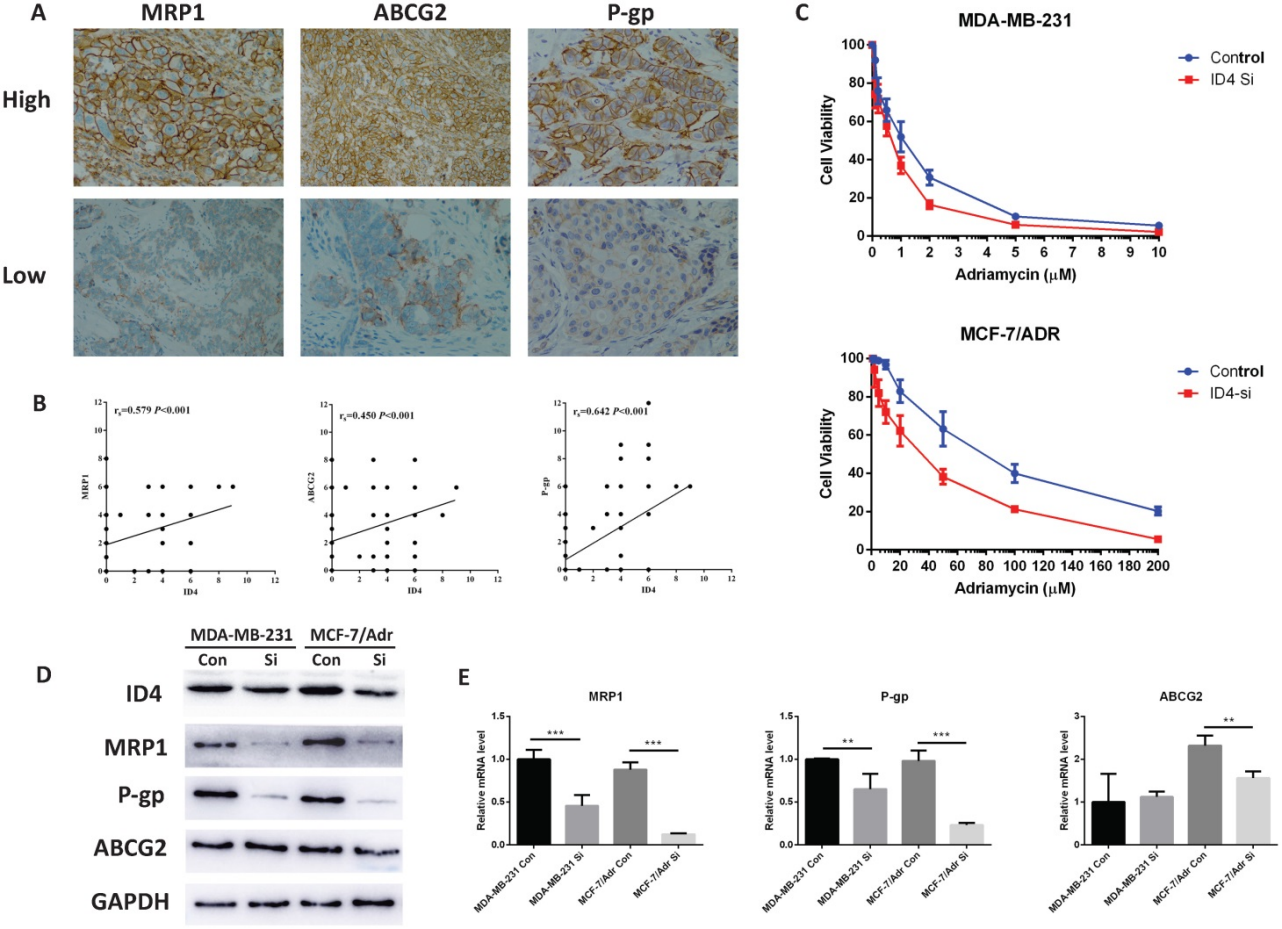
** ID4 was correlated with chemo-resistance related proteins. (A)** Representative immunostaining of MRP1, ABCG2 and P-gp in human breast cancer samples. All of the three proteins immunoreactivity were detected on the cell membrane. Magnifications were 200× in ABCG2 high and MRP1 low cases, and 400× in other cases. **(B)** IHC assay showed that ID4 expression was positively correlated with the expression of MRP1 (r=0.579, P<0.001), ABCG2 (r=0.450, P<0.001) and P-gp (r=0.642, P<0.001). **(C)** Down regulation of ID4 increased the sensitivity of Adriamycin in breast cancer cell lines. The protein **(D)** and mRNA **(E)** levels of the MRP1, P-gp and ABCG2 were measured by Western Blotting and qRT-PCR analyses respectively. GAPDH was used as an internal control. The results represent mean ± s.d. from three independent experiments. *P< 0.05 vs. NC, **P< 0.01 vs. NC, ***P < 0.001 vs. NC.

As mentioned before, ID is involved in chemo-resistance in several tumors, and multidrug resistance was regarded as the greatest obstacle in TNBC therapy. We proposed a relationship between ID4 and triple negative breast cancer chemo- resistance. To confirm this hypothesis, we first tested the expression of ID4 and some drug resistant related ABC transporter proteins by Immunohistochemistry (Fig. 4A). The results showed that there was positive correlation between ID4 and MRP1, ABCG2, P-gp (Fig. 4B). To further investigate the function of ID4 in breast cancer chemo-resistance, we performed conventional CCK8 assays to identify the sensitivity of Adriamycin in breast cancer cells. The IC_50_ values of Adriamycin were significantly decreased in ID4 knockdown MCF-7/Adr and MDB-MA-231 cell lines (Fig. 4C). Furthermore, we detected the expression of ABC transporter proteins in protein and mRNA levels by using Western-blotting and qRT-PCR. A positive correlation between the expression of ID4 and MRP1, P-gp was observed in both TNBC cell lines, and ABCG2 decreased in ID4 knockdown MCF-7/Adr with no change in MDA-MB-231 ID4 knockdown cells.

### ID4 is associated with breast cancer chemo-resistance via CBF1/MRP1 signaling

**Figure 5:**
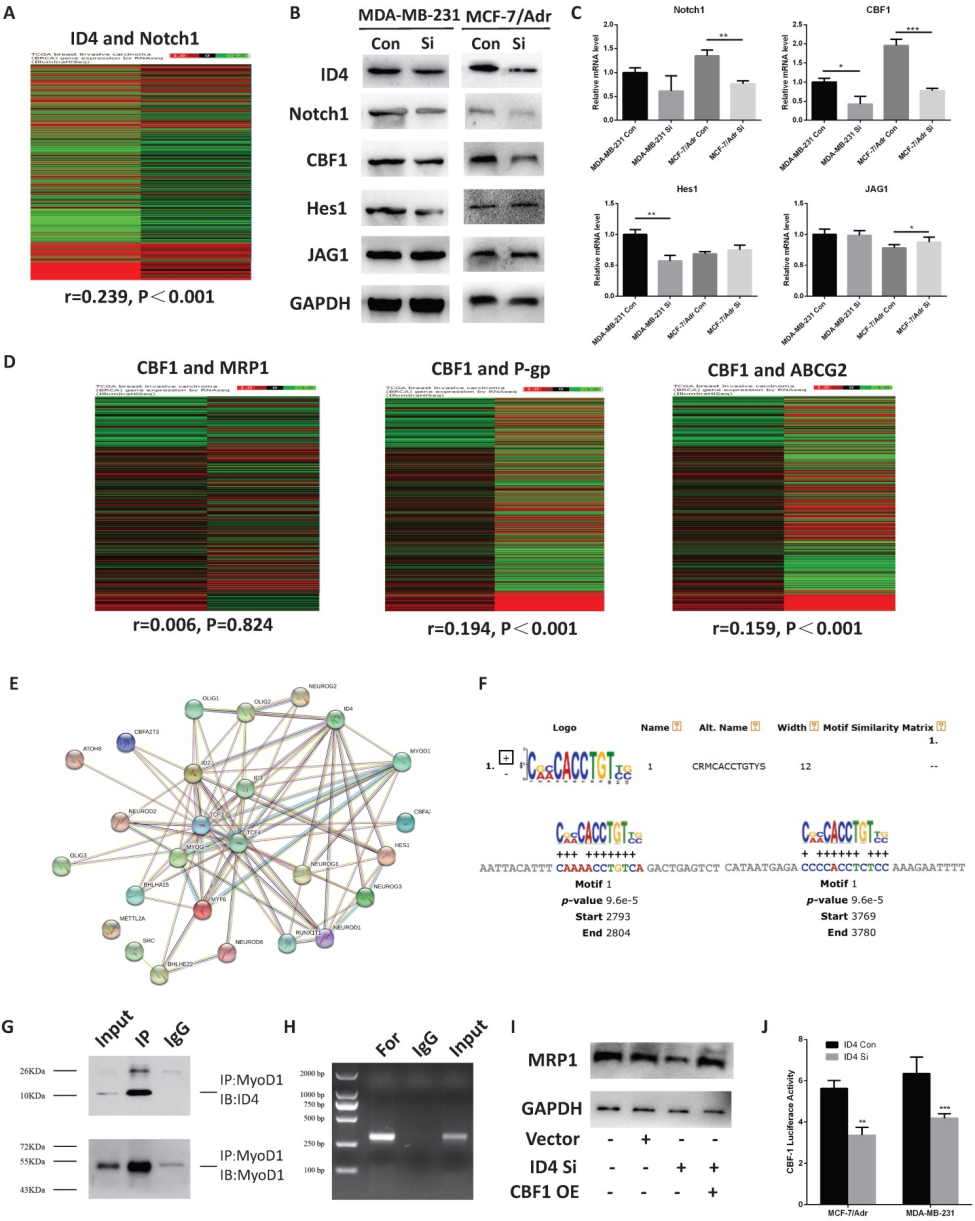
** ID4 was correlated with Notch1 pathway. (A)** TCGA database showed that ID4 was positively correlated with Notch1 (r=0.239, P<0.001) expression in tumor tissue samples. n=1214. The protein **(B)** and mRNA **(C)** levels of the Notch1, CBF1, Hes1 and JAG1 were measured by Western Blotting and qRT-PCR analyses respectively. GAPDH was used as an internal control. The results represent mean ± s.d. from three independent experiments. *P< 0.05 vs. NC, **P< 0.01 vs. NC, ***P < 0.001 vs. NC. **(D)** TCGA database showed that ID4 was positively correlated with P-gp (r=0.194, P<0.001) and ABCG2 (r=0.159, P<0.001) expression in tumor tissue samples. n=1214. **(E)** STRING Interaction Network searching result on Genecards website showed ID4 could be interacted with MyoD1. **(F)** MEME database database results indicated that, CRMCACCTGTYS, the motif of MyoD1 could combine at -7196 to -7207 and-6220 to -6231 sites of CBF1 TSS. **(G)** Co-IP assays confirmed that ID4 could combine with MyoD1 in MCF7/Adr cells. **(H)** CHIP results showed MyoD1 could directly bind to the promoter region of CBF1 in MCF7/Adr cells. **(I)** Western blotting results showed the upregulation of CBF1 could increase the expression of MRP1 after knockdown ID4 in MCF7/Adr cells.

To further investigate the potential function of ID4 in chemo-resistance, we analyze the TCGA database and found that the expression of ID4 correlated with the Notch1 pathway in breast cancer (Fig. 5A). Furthermore, we detected the expression of Notch1 pathway related proteins in protein and mRNA levels by using Western-blotting and qRT- PCR. A positive correlation between the expression of ID4 and C-promoter binding factor-1(CBF1, a critical downstream transcriptional factor in Notch1 signal pathway) was observed in both TNBC breast cancer cell lines,(Fig. 5B, 5C). Interestingly, we found (through the TCGA database) that CBF1 expression positively correlated with the P-gp and ABCG2 but not with MRP1(Fig. 5D). Though ID4 was considered as a negative transcription factor, it could not directly bind to promoter region due to lacking DNA binding domain. Thus, it was widely reported that ID4 could perform its function by inhibiting other transcription factors through HLH domain. We found MyoD1, which was recently reported to performing as a transcriptor repressor in breast cancer, was the exclusive factor could be combined with both ID4 and CBF1 from STRING Interaction Network (https:// version11.string-db.org/cgi/network.pl?taskId=7z99iaiuOrw8) and Top Transcription factor binding sites by Qiagen on Genecards website (https://www.genecards.org/cgi-bin/carddisp.pl?gene=id4), respectively. And then we used footprintDB database (http://floresta. eead.csic.es/footprintdb/index.php) to find two motifs of MyoD1, CRMCACCTGTYS and SCASCTGTY, and the 10kb upstream sequences of CBF1 TSS were used to confirm the binding region of the two motifs of MyoD1 by MEME database (http://meme-suite.org/tools/mast). The results showed that CRMCACCTGTYS could combine at -7196 to -7207 and-6220 to -6231 sites (TSS, Fig 5E) but no corresponding binding sites were found for SCASCTGTY. To confirm this hypothesis, we performed a Co-IP assay and the results show that ID4 could combine with MyoD1 (Fig. 5F), suggesting that ID4, which was always acting as a negative transcription factor, might regulate downstream genes by inhibiting function of MyoD1. Afterwards, the CHIP assay results afterwards showed that MyoD1 may directly bind to the promoter region of CBF1 (Fig 5G). As MyoD1 was reported as a negative regulator in breast cancer, these evidences indicated that ID4 could promoted the expression of CBF1 by weakening the inhibition of MyoD1 on CBF1 transcription in breast cancer (Fig. 5H). In addition, the expression of MRP1 increased when we overexpressed CBF1 after knockdown ID4 (Fig. 5I), suggesting ID4 may regulate the expression of MRP1 through CBF1.

### Knockdown of ID4 suppressed tumor growth and enhanced Adriamycin sensitivity *in vivo*

**Figure 6:**
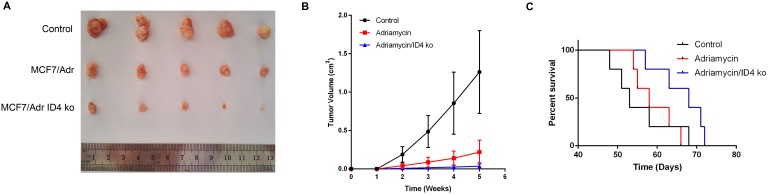
** Effects of ID4 on tumorigenic and chemo-resistant capacities of the breast cancer cells *in vivo*. (A)** Representative xenograft tumors at 5weeks after inoculation. **(B)** Growth curves of xenograft tumors were shown. Data were presented as mean ± s.d. from three independent experiments. **(C)** Curve graph indicated survival time of the xenograft mice.

Considering the *in vitro* involvement of ID4 in breast cancer cell proliferation and chemo-resistance, we extended this study to determine the impact of ID4 on tumorigenic capabilities of breast cancer cells *in vivo*. When the MCF-7/Adr cells (transduced with the GV248 lentiviral vectors) expressing shRNA targeting ID4 or non-targeting controls were subcutaneously implanted into the immunocompromised mice, we observed a significant decrease in tumor formation and an increase in Adriamycin sensitivity and the growth of tumor bearing mice when ID4 was inhibited (Fig 6A, 6B, 6C).

## Discussion

Breast cancer is the most common invasive cancer and accounts for approximately 30% of all new cancer diagnoses in women 1. Despite significant progress in tumorigenesis and treatment strategies, resistance to chemotherapeutic agents remains a consistent obstacle in terms of treatment success, especially for TNBC 3. ID (inhibitor of DNA binding) factors (ID1-ID4) contain a highly conserved helix-loop-helix dimerization domain through which they form heterodimers with basic helix-loop-helix transcription factors 26. It is reported that IDs are involved in numerous cell processes, including cell proliferation, differentiation, and tumorigenesis. ID4 is a newly discovered member in the ID family involved in various cellular processes in cancer. Sometimes it is an oncogene. Its over-expression promotes the occurrence and development of tumors such as astrocytoma and bladder cancer 27-30. It is also regarded as a tumor suppressor gene because the methylation of ID4 promoter gene leading to ID4 silencing can also promote the development of colon cancer, gastric cancer, prostate cancer and hematological malignancies 31-34. Therefore, ID4 may become an important index for judging the prognosis of tumors and a potential target for cancer therapy.

In breast cancer, ID4 is considered as the key controller of luminal differentiation pathways, and ID4 expression is almost absent in ERα breast cancer cells, indicating that ERα might negatively regulate ID4 function. In basal-like breast cancers on the other hand, ID4 is overexpressed and related with poor prognosis and a stem-like transcriptional profile. However, meta-analysis did not show any significant association between ID4 and breast cancer. In this study, we firstly confirmed that ID4 was highly expressed in the TNBC and Her-2 overexpression breast cancers, and its expression was negative correlated with ER and PR, which was consistent with Best and Garraway's reports. In addition, we demonstrated that ID4 knockdown could inhibit the proliferation, invasion of MDA-MB-231 and MCF7/ Adr cells, confirming ID4 acting as an aggressive promoter in TNBC.

Recent research mentioned that ID4 may be involved in the chemo-resistance process. Jeon reported that ID4 drive drug resistance of glioma cells by miR-9-SOX2-ABCC3/ABCC6 regulatory pathway. ID4 induced non-small lung cancer cells rejected to cisplatin with activated p38-MAPK pathway. Otherwise, no evidence showed that the mechanism of ID4 influenced chemo-resistance in TNBC. In this work, we choose MCF-7/Adr (Adriamycin-resistant TNBC cell lines) and MDA-MB-231 (TNBC cell lines) to observe whether ID4 is involved in the chemo- resistance of TNBC. Firstly, we found that the expression of ID4 was associated with chemo-resistant ABC transporter proteins, including MRP1, ABCG2, P-gp in breast cancer resection samples. Moreover, both breast cells became more sensitive to Adriamycin after ID4 knockdown and the finding was verified *in vivo*.

In order to further explore the regulatory mechanism of ID4 on MRP1, we used TCGA to analyze the main downstream signaling pathways of ID4 in breast cancer and found that Notch1 pathway has been involved in many development processes. In our work, we found the Notch1 pathway was positively associated with ID4 expression both in MDA-MB-231 and MCF-7/Adr cells, and ID4 could activate CBF1 by directly binding to the CBF1 promoter region via combined with MyoD1, which was reported to be a negative transcriptor in breast cancer 35. Recently, Cho et al reported that CBF1 could up-regulate the expression of MRP1 directly 36. We tested to detect whether CBF1 was involved in the MRP1 regulation in our research: When we stimulated CBF1 expression after knock-down of ID4, the expression of MRP1 was significantly higher than the control, suggesting that CBF1 could be involved in the regulation of MRP1 expression. Considering the above results, we believed that regulation of MRP1 expression by ID4 is mediated by CBF1. In our data, expression of P-gp decreased both in ID4 knockdown MCF-7/Adr and MDA-MB-231 cells, and there was also a positive correlation between CBF1 expression and P-gp, on TCGA database. The results indicated ID4 may not only control chemotherapy resistance through CBF1- MRP1 pathway, but can also via CBF1 involved P-gp regulation. These require further research.

In summary, we have for the first time provided unequivocal evidence demonstrating that ID4 performs its function in part via regulating breast cancer chemo-resistance through the ID4-CBF1-MRP1 axis. These novel findings can provide a new perspective for mammary carcinoma chemotherapeutic intervention.

## Figures and Tables

**Table 1:** Oligonucleotide primers used for qRT-PCR.

	Forward Primer	Reverse Primer
**ID4**	5′-GTGCGATATGAACGACTGCT-3′	5′-CAGGATCTCCACTTTGCTGA-3′
**MRP1**	5′-TTCTAGTGTTGGACGAGGCT-3'	5′-TGGCCATGCTATA-3'
**P-gp**	5'-CCCATCATTGCAATAGCAGG-3'	5'3GTTCAAACTTCTGCTCCTGA-3'
**ABCG2**	5′-TGCCCAGGACTCAATGCAACAG-3′	5′-GACTGAAGGGCTACTAACC-3′.
**Notch1**	5'-AAGCTGCATCCAGAGGCAAAC-3′	5'-TGGCATACACACTCCGAGAACAC-3'
**CBF1**	5'-AATCCCGGAGTCAACATGC-3'	5'-TCTCATCTTGAAAAGCCAACG-3'
**Hes1**	5'-GGACATTCTGGAAATGACAGTGA-3'	5'-AGCACACTTGGGTCTGTGCTC-3'​
**JAG1**	5ʹ-CTCATCAGCGGTGTCTCAAC-3ʹ	5ʹ-GGCACACAC ACTTAAATCCG-3ʹ
**GAPDH**	5'-CGTATTGGGCGCCTGGTCAC-3'	5'-ATGATGACCCTTTTGGCTCC-3'

## References

[1] Siegel RL, Miller KD, Jemal A (2019). Cancer statistics, 2019. CA Cancer J Clin.

[2] Ahn SG, Kim SJ, Kim C, Jeong J (2016). Molecular Classification of Triple-Negative Breast Cancer. J Breast Cancer.

[3] Lee A, Djamgoz MBA (2018). Triple negative breast cancer: Emerging therapeutic modalities and novel combination therapies. Cancer Treat Rev.

[4] Lehmann BD, Bauer JA, Chen X, Sanders ME, Chakravarthy AB, Shyr Y (2011). Identification of human triple-negative breast cancer subtypes and preclinical models for selection of targeted therapies. J Clin Invest.

[5] Elias AD (2010). Triple-negative breast cancer: a short review. Am J Clin Oncol.

[6] Moiseenko F, Volkov N, Bogdanov A, Dubina M, Moiseyenko V (2017). Resistance mechanisms to drug therapy in breast cancer and other solid tumors: An opinion. F1000Res.

[7] Abotaleb M, Kubatka P, Caprnda M, Varghese E, Zolakova B, Zubor P (2018). Chemotherapeutic agents for the treatment of metastatic breast cancer: An update. Biomed Pharmacother.

[8] Perk J, Iavarone A, Benezra R (2005). Id family of helix-loop-helix proteins in cancer. Nat Rev Cancer.

[9] Lasorella A, Benezra R, Iavarone A (2014). The ID proteins: master regulators of cancer stem cells and tumour aggressiveness. Nat Rev Cancer.

[10] Patel D, Morton DJ, Carey J, Havrda MC, Chaudhary J (2015). Inhibitor of differentiation 4 (ID4): From development to cancer. Biochimica et biophysica acta.

[11] Jeon HM, Jin X, Lee JS, Oh SY, Sohn YW, Park HJ (2008). Inhibitor of differentiation 4 drives brain tumor-initiating cell genesis through cyclin E and notch signaling. Genes Dev.

[12] Chan F, Oatley MJ, Kaucher AV, Yang QE, Bieberich CJ, Shashikant CS (2014). Functional and molecular features of the Id4+ germline stem cell population in mouse testes. Genes Dev.

[13] Roldan G, Delgado L, Muse IM (2006). Tumoral expression of BRCA1, estrogen receptor alpha and ID4 protein in patients with sporadic breast cancer. Cancer biology & therapy.

[14] Nasif D, Campoy E, Laurito S, Branham R, Urrutia G, Roque M (2018). Epigenetic regulation of ID4 in breast cancer: tumor suppressor or oncogene?. Clin Epigenetics.

[15] Baker LA, Holliday H, Swarbrick A (2016). ID4 controls luminal lineage commitment in normal mammary epithelium and inhibits BRCA1 function in basal-like breast cancer. Endocr Relat Cancer.

[16] Thike AA, Tan PH, Ikeda M, Iqbal J (2016). Increased ID4 expression, accompanied by mutant p53 accumulation and loss of BRCA1/2 proteins in triple-negative breast cancer, adversely affects survival. Histopathology.

[17] Branham MT, Campoy E, Laurito S, Branham R, Urrutia G, Orozco J (2016). Epigenetic regulation of ID4 in the determination of the BRCAness phenotype in breast cancer. Breast Cancer Res Treat.

[18] Fontemaggi G, Dell'Orso S, Trisciuoglio D, Shay T, Melucci E, Fazi F (2009). The execution of the transcriptional axis mutant p53, E2F1 and ID4 promotes tumor neo-angiogenesis. Nat Struct Mol Biol.

[19] Qi K, Li Y, Li X, Lei X, Wang B, Zhang L (2016). Id4 promotes cisplatin resistance in lung cancer through the p38 MAPK pathway. Anticancer Drugs.

[20] Dong J, Huang S, Caikovski M, Ji S, McGrath A, Custorio MG (2011). ID4 regulates mammary gland development by suppressing p38MAPK activity. Development.

[21] Jeon HM, Sohn YW, Oh SY, Kim SH, Beck S, Kim S (2011). ID4 imparts chemoresistance and cancer stemness to glioma cells by derepressing miR-9*-mediated suppression of SOX2. Cancer research.

[22] Farnie G, Clarke RB (2007). Mammary stem cells and breast cancer-role of Notch signalling. Stem cell reviews.

[23] Sajadimajd S, Yazdanparast R (2017). Sensitizing effect of juglone is mediated by down regulation of Notch1 signaling pathway in trastuzumab-resistant SKBR3 cells. Apoptosis: an international journal on programmed cell death.

[24] Chiba S (2006). Notch signaling in stem cell systems. Stem cells.

[25] Yoon K, Gaiano N (2005). Notch signaling in the mammalian central nervous system: insights from mouse mutants. Nature neuroscience.

[26] Zhang R, Boareto M, Engler A, Louvi A, Giachino C, Iber D (2019). Id4 Downstream of Notch2 Maintains Neural Stem Cell Quiescence in the Adult Hippocampus. Cell Rep.

[27] Rahme GJ, Israel MA (2015). Id4 suppresses MMP2-mediated invasion of glioblastoma-derived cells by direct inactivation of Twist1 function. Oncogene.

[28] Martini M, Cenci T, D'Alessandris GQ, Cesarini V, Cocomazzi A, Ricci-Vitiani L (2013). Epigenetic silencing of Id4 identifies a glioblastoma subgroup with a better prognosis as a consequence of an inhibition of angiogenesis. Cancer.

[29] Garcia-Baquero R, Puerta P, Beltran M, Alvarez M, Sacristan R, Alvarez-Ossorio JL (2013). Methylation of a novel panel of tumor suppressor genes in urine moves forward noninvasive diagnosis and prognosis of bladder cancer: a 2-center prospective study. The Journal of urology.

[30] Garcia-Baquero R, Puerta P, Beltran M, Alvarez-Mujica M, Alvarez-Ossorio JL, Sanchez-Carbayo M (2014). Methylation of tumor suppressor genes in a novel panel predicts clinical outcome in paraffin-embedded bladder tumors. Tumour biology: the journal of the International Society for Oncodevelopmental Biology and Medicine.

[31] Umetani N, Takeuchi H, Fujimoto A, Shinozaki M, Bilchik AJ, Hoon DS (2004). Epigenetic inactivation of ID4 in colorectal carcinomas correlates with poor differentiation and unfavorable prognosis. Clinical cancer research: an official journal of the American Association for Cancer Research.

[32] Chan AS, Tsui WY, Chen X, Chu KM, Chan TL, Chan AS (2003). Downregulation of ID4 by promoter hypermethylation in gastric adenocarcinoma. Oncogene.

[33] Patel D, Knowell AE, Korang-Yeboah M, Sharma P, Joshi J, Glymph S (2014). Inhibitor of differentiation 4 (ID4) inactivation promotes de novo steroidogenesis and castration-resistant prostate cancer. Molecular endocrinology.

[34] Yu L, Liu C, Vandeusen J, Becknell B, Dai Z, Wu YZ (2005). Global assessment of promoter methylation in a mouse model of cancer identifies ID4 as a putative tumor-suppressor gene in human leukemia. Nature genetics.

[35] Zhang Q, Liu XY, Li S, Zhao Z, Li J, Cui MK (2017). Repression of ESR1 transcription by MYOD potentiates letrozole-resistance in ERalpha-positive breast cancer cells. Biochem Biophys Res Commun.

[36] Cho S, Lu M, He X, Ee PL, Bhat U, Schneider E (2011). Notch1 regulates the expression of the multidrug resistance gene ABCC1/MRP1 in cultured cancer cells. Proceedings of the National Academy of Sciences of the United States of America.

